# Biomechanics and lower limb function are altered in athletes and runners with achilles tendinopathy compared with healthy controls: A systematic review

**DOI:** 10.3389/fspor.2022.1012471

**Published:** 2023-01-04

**Authors:** Andrew Quarmby, Jamal Mönnig, Hendrik Mugele, Jakob Henschke, MyoungHwee Kim, Michael Cassel, Tilman Engel

**Affiliations:** ^1^ University Outpatient Clinic, Sports Medicine & Sports Orthopaedics, University of Potsdam, Potsdam, Germany; ^2^ Department of Sport Science, Laboratory for Environmental and Exercise Science, University of Innsbruck, Innsbruck, Austria

**Keywords:** achilles tendinopathy, biomechanics, neuromuscular, kinetics, electromyography, athletes, runners, kinematics

## Abstract

Achilles tendinopathy (AT) is a debilitating injury in athletes, especially for those engaged in repetitive stretch-shortening cycle activities. Clinical risk factors are numerous, but it has been suggested that altered biomechanics might be associated with AT. No systematic review has been conducted investigating these biomechanical alterations in specifically athletic populations. Therefore, the aim of this systematic review was to compare the lower-limb biomechanics of athletes with AT to athletically matched asymptomatic controls. Databases were searched for relevant studies investigating biomechanics during gait activities and other motor tasks such as hopping, isolated strength tasks, and reflex responses. Inclusion criteria for studies were an AT diagnosis in at least one group, cross-sectional or prospective data, at least one outcome comparing biomechanical data between an AT and healthy group, and athletic populations. Studies were excluded if patients had Achilles tendon rupture/surgery, participants reported injuries other than AT, and when only within-subject data was available.. Effect sizes (Cohen's *d*) with 95% confidence intervals were calculated for relevant outcomes. The initial search yielded 4,442 studies. After screening, twenty studies (775 total participants) were synthesised, reporting on a wide range of biomechanical outcomes. Females were under-represented and patients in the AT group were three years older on average. Biomechanical alterations were identified in some studies during running, hopping, jumping, strength tasks and reflex activity. Equally, several biomechanical variables studied were not associated with AT in included studies, indicating a conflicting picture. Kinematics in AT patients appeared to be altered in the lower limb, potentially indicating a pattern of “medial collapse”. Muscular activity of the calf and hips was different between groups, whereby AT patients exhibited greater calf electromyographic amplitudes despite lower plantar flexor strength. Overall, dynamic maximal strength of the plantar flexors, and isometric strength of the hips might be reduced in the AT group. This systematic review reports on several biomechanical alterations in athletes with AT. With further research, these factors could potentially form treatment targets for clinicians, although clinical approaches should take other contributing health factors into account. The studies included were of low quality, and currently no solid conclusions can be drawn.

## 1. Introduction

Achilles tendinopathy (AT) is a debilitating overuse injury, symptoms of which can include pain localized to the Achilles tendon, morning stiffness, and functional impairments during dynamic activities such as running and hopping (1, 2). Achilles tendinopathy is a recurrent problem for both athletic and non-athletic populations (3–5). Whilst a study by Lysholm et al. (6), reported a 9% annual incidence of Achilles disorders in runners, a different investigation found an AT point prevalence of 36% in approximately 1,000 runners (7). A separate study by Albers et al. (4), found that 65% of AT cases do not involve sport. Current research therefore indicates that mechanisms of AT development might be multi-factorial in nature and injury presentation may differ according to population category i.e., athletic vs. non-athletic (3).

Clinical risk factors for AT have been discussed in a recent publication by van der Vlist et al. (5), and include prior lower limb tendinopathy or fracture, use of ofloxacin antibiotics, moderate alcohol consumption, increased time between heart transplantation and initiation of treatment for infectious disease, as well as cold weather training. Furthermore, various neuromechanical indications relating to human biomechanics seem to increase the risk of AT. These neuromechanical factors may be manifested in decreased isokinetic plantar flexor strength, and abnormal gait pattern with decreased forward progression of propulsion and more lateral foot-roll over at the forefoot flat phase (5). Such factors may be of particular relevance for athletic populations, especially for those engaged in activities that require repetitive stretch-shortening-cycle loading (SSC), such as running and jumping (2, 8, 9). It has been hypothesized that repetitive loading of the Achilles tendon, which is not compensated *via* sufficient strength or endurance of the plantar flexor muscles or optimal gait biomechanics, may result in injury (1, 2, 5, 9–11). This has led to the widespread implementation of biomechanically-driven and strength-based loading programs in the rehabilitation and prevention of AT (12–16). However, it is important to understand this model in the context of other potential contributing factors to AT overuse injury, such as increasing age (17), training load (18), increased BMI (19) and other considerations mentioned previously (5).

Several systematic reviews and meta-analyses have recently investigated the relationship between biomechanical factors and AT (2, 10, 20, 21). In two independent meta-analyses, Hasani et al. (21), and McAuliffe et al. (10), concluded plantar-flexor strength deficits to be associated with AT, when compared within-subject (affected vs. healthy limb) or with healthy controls. Although, deficits were more pronounced between sides than when compared with the control group in the more recent analysis (21). Two further systematic reviews focused on aspects of gait and lower-limb biomechanics (2, 20). Sancho et al. (2019) (2), reported biomechanical alterations in AT patients during running and hopping after conducting a meta-analysis across 16 studies, including changes in kinematics, kinetics and muscle activity. A similar review including 14 studies found comparable results regarding alterations in gait in AT patients (20). It should be noted that both reviews indicated a high risk of bias across studies and recognized a lack of high-quality prospective research in the area. Another interesting avenue of enquiry is adaptations of reflex responses in patients with AT, and two prominent studies have suggested higher volitional supraspinal reflexes (22) and altered central nervous system reflex regulation in tendinopathic tendons (23). Considering altered reflex responses have been observed in other persistent musculoskeletal pain disorders (24), their relevance for AT patients may warrant further exploration and review.

As described, a range of data summarised in multiple studies has revealed weak to moderate evidence that the biomechanics of patients with AT are potentially altered (2, 10, 20, 21). However, these reviews have tended to focus on a single component of human movement e.g., isolated joint strength (10, 21) or gait mechanics (2, 20), providing a useful but arguably narrower picture of the data. Thus, synthesising the evidence into a single comprehensive review could prove helpful in furthering understanding of these alterations in AT populations. Besides this, none of the above-mentioned reviews implemented a set training load within inclusion criteria e.g., running >20 km per week or equivalent, even though clinical presentation of AT may vary between athletic and non-athletic populations (3, 5). In addition, three of the reviews included studies which compared parameters associated with AT between sides within the injured group (affected vs. healthy limb) (2, 10, 21), despite evidence suggesting that the contra-lateral healthy limb might also present with sensory motor deficits in tendinopathy patients (25) and research indicating central sensitization and altered central pain processing in AT (26, 27). Furthermore, in two of the previously conducted reviews there was no set criteria for AT diagnosis stated within the inclusion criteria of investigated studies (10, 20), although best practice diagnosis guidelines have previously been outlined (28, 29).

Therefore, the aim of this study was to conduct a systematic review, with the goal of synthesising information regarding biomechanical alterations and changes in lower limb function in specifically athletic populations with AT, when compared to an asymptomatic, athletic, healthy control group. Populations in both groups were defined as athletic, based upon strict inclusion criteria.

## 2. Methods

This systematic review was conducted and reported in accordance with the PRISMA (Preferred Reporting Items for Systematic Reviews and Meta-Analyses) guidelines (30). The review was not pre-registered.

### 2.1. Search strategy

The electronic databases MEDLINE, Web of Science and Cochrane Library were searched in March 2021. Two authors (A.Q., J.M.) completed the initial search of all databases simultaneously, after critical discussion of key terms and development of a search strategy. The four categories identified as the base of the search strategy were “Biomechanics”, “Movement Task”, “Pathology (Tendinopathy)” and “Anatomical Location (Achilles tendon)”. MeSH terms were also applied to enabled search terms and in databases which featured this function. Filters of (1) Human subjects/Not animals, (2) Language: Only English or German articles and (3) Research published in the last 20 years (2001–2021) were applied either directly as search terms or within filter settings of the corresponding database. Search terms for MEDLINE (PubMed) are detailed in Table 1.

**Table 1:** MEDLINE (PubMed) search strategy.

Category	Terms
Biomechanics	(“Biomechanical phenomena"[MeSH]) OR (“Biomechanic*"[Title/Abstract]) OR (“Kinematic*"[Title/Abstract]) OR (“Kinetic*"[Title/Abstract]) OR (“Kinetics"[MeSH]) OR (“Motion"[MeSH]) OR (“Temporospatial"[Title/Abstract]) OR (“Plantar pressure"[Title/Abstract]) OR (“Ground reaction force"[Title/Abstract]) OR (“GRF"[Title/Abstract]) OR (“Moment"[Title/Abstract]) OR (“Torque"[MeSH]) OR (“Force"[Title/Abstract]) OR (“Stiffness"[Title/Abstract]) OR (“3D Kinematics"[Title/Abstract]) OR (“Mechanic*"[Title/Abstract]) OR (“Mechanics"[MeSH]) OR (“Muscular"[Title/Abstract]) OR (“Neuromuscular"[Title/Abstract]) OR (“Neuro-muscular"[Title/Abstract]) OR (“Neuromotor"[Title/Abstract]) OR (“Neuromotor control"[Title/Abstract]) OR (“Motor control"[Title/Abstract]) OR (“Reflex” [MeSH]) OR (“Reflex, stretch"[MeSH]) OR (“EMG"[Title/Abstract]) OR (“Electromyograph*"[Title/Abstract]) OR (“Electromyography"[MeSH]) OR (“Muscle activit*"[Title/Abstract]) OR (“Strength*"[Title/Abstract]) OR (“Muscle strength"[MeSH]) OR (“Weak*"[Title/Abstract]) OR (“Strong*"[Title/Abstract]) OR (“Power*"[Title/Abstract]) OR (“Muscle*"[Title/Abstract]) OR (“Muscles"[MeSH]) OR (“Function*"[Title/Abstract]) OR (“Endurance"[Title/Abstract]) OR (“Fatigue*"[MeSH]) OR (“Muscle fatigue"[MeSH]) OR (“Stiff*"[Title/Abstract]) OR (“Rate of force development"[Title/Abstract]) OR (“RFD"[Title/Abstract]) OR (“Stress"[Title/Abstract]) OR (“Strain"[Title/Abstract])
Movement Task	(“Running"[MeSH]) OR (“Walking"[MeSH]) OR (“Gait"[MeSH]) OR (“Running Gait"[Title/Abstract]) OR (“Gait-related” [Title/Abstract]) OR (“Gait related” [Title/Abstract]) OR (“Locomotion"[MeSH]) OR (“Bounc*"[Title/Abstract]) OR (“Plyometric*"[Title/Abstract]) OR (“Plyometric exercise"[MeSH]) OR (“Jump*"[Title/Abstract]) OR (“Hopping"[Title/Abstract]) OR (“Hop"[Title/Abstract]) OR (“Land*"[Title/Abstract]) OR (“Drop*"[Title/Abstract]) OR (“Isokinetic*"[Title/Abstract]) OR (“Concentric*"[Title/Abstract]) OR (“Eccentric*"[Title/Abstract]) OR (“Isometric*"[Title/Abstract]) OR (“Isometric contraction"[MeSH]) OR (“Resistance Training"[MeSH])
Pathology (Tendinopathy)	(“Tendinopathy"[MeSH]) OR (“Achilles Tendinopathy"[Title/Abstract]) OR (“Tendinitis"[Title/Abstract]) OR (“Tendinosis"[Title/Abstract])
Anatomical Location (Achilles tendon)	(“Achilles tendon"[MeSH]) OR (“Plantarflex*"[Title/Abstract])
(Human Subjects)	NOT (Animals)

The categories were combined using the Boolean command “AND”.

### 2.2. Eligibility criteria

Study eligibility was determined based upon strict inclusion and exclusion criteria, which were defined as follows:

Inclusion Criteria

• AT diagnosis based upon established guidelines – History of localised Achilles tendon pain (mid-portion and/or insertional), and at least one of the following: pain during or after activities that load the tendon, morning stiffness, and tenderness on palpation.
• Data should be cross-sectional, prospective or baseline data from intervention studies.
• Studies comparing biomechanical features during human gait, in hopping, jumping or other functional movement activity, during isolated strength activities or measuring reflex activity between AT patients and healthy asymptomatic controls.
• Population should be an athletic/recreational athletic population in regular training e.g., >20 km running/training >2 h a week. Sport should include repetitive SSC load on Achilles tendon.
• Articles in English or German.

Exclusion Criteria

• Participants with Achilles tendon rupture and/or surgical intervention.
• Studies including participants with injury other than Achilles tendinopathy.
• Reviews, case-series, case studies, opinion articles and abstracts.
• Studies comparing within-subject e.g., injured vs. non-injured leg.

### 2.3. Selection process

All studies were screened independently by two of the authors (A.Q., J.M.). Titles and abstracts of all obtained records were downloaded into an electronic reference management software (Mendeley Desktop 1.19.4). Duplicates were removed with the aid of the automatic detection system within the reference manager and manually checked. Titles and abstracts of studies were matched against pre-defined inclusion and exclusion criteria for eligibility. Articles included by title and abstract were then assessed for inclusion by full text, and if no reason for exclusion was discovered the articles were included for synthesis within the systematic review. Any disagreements on study inclusion or exclusion were discussed and resolved between the two authors (A.Q., J.M.) in conversations arbitrated by a third author (T.E.). The reference lists of included studies and relevant systematic reviews were also searched to look for potential studies that might meet inclusion criteria.

### 2.4. Risk of bias assessment

The risk of bias for included studies was assessed using the Critical Skills Appraisal Programme Case-Control Study Checklist (31). The original checklist contains 12 questions but only 10 were relevant to the studies within this systematic review. Therefore, these 10 questions were applied to assess the quality of the included studies, as adapted previously in a similar systematic review (10). A list of the questions, their associated criteria and scoring strategy are provided in Supplemental File S1. The risk of bias assessment was performed independently by two authors (A.Q., J.M.). Disagreements on scoring of the individual studies were managed by consensus and if agreement could not be reached, a third author (T.E.) was consulted to resolve the debate.

### 2.5. Data extraction and analysis

A pre-defined data extraction sheet was prepared with the following variables: sample size, participant demographics and details (e.g., age, sex, anthropometrics, training status, AT diagnosis and symptoms duration), study design, characteristics of the task investigated, biomechanical variables studied and any reported significant findings. Data were extracted by each reviewer (A.Q., J.M.) for the included studies. Study results were then sub-categorised by the task characteristics investigated, to allow for a synthesis of variables within each pre-defined area of motor behaviour. Task characteristics were categorised as (1) “Gait” – running/walking, (2) “Non-gait multi-joint activity” – hopping/jumping/squatting, (3) “Isolated joint strength”, (4) “Reflex activity” – specific methodologies targeting reflex responses. Relevant biomechanical variables associated with the specific movement behaviour, were then reported within each category. In cases where studies reported on more than one relevant task, findings from the single study were extracted, separated, and binned into the appropriate category. In cases where data needed for inclusion was not found within the manuscript, the relevant authors were contacted to obtain the specific details required. When available, means and standard deviations were extracted from included articles. These were used to calculate effect sizes (Cohen's *d*) with corresponding 95% confidence intervals, allowing for better comparison between the studies (reported as: Effect Size (ES) *d* [95% Confidence Intervals (CIs)]). Effect sizes were considered statistically significant (indicated with*), if 95% confidence intervals did not cross the zero level.

## 3. Results

### 3.1. Identification of studies

**Figure 1:**
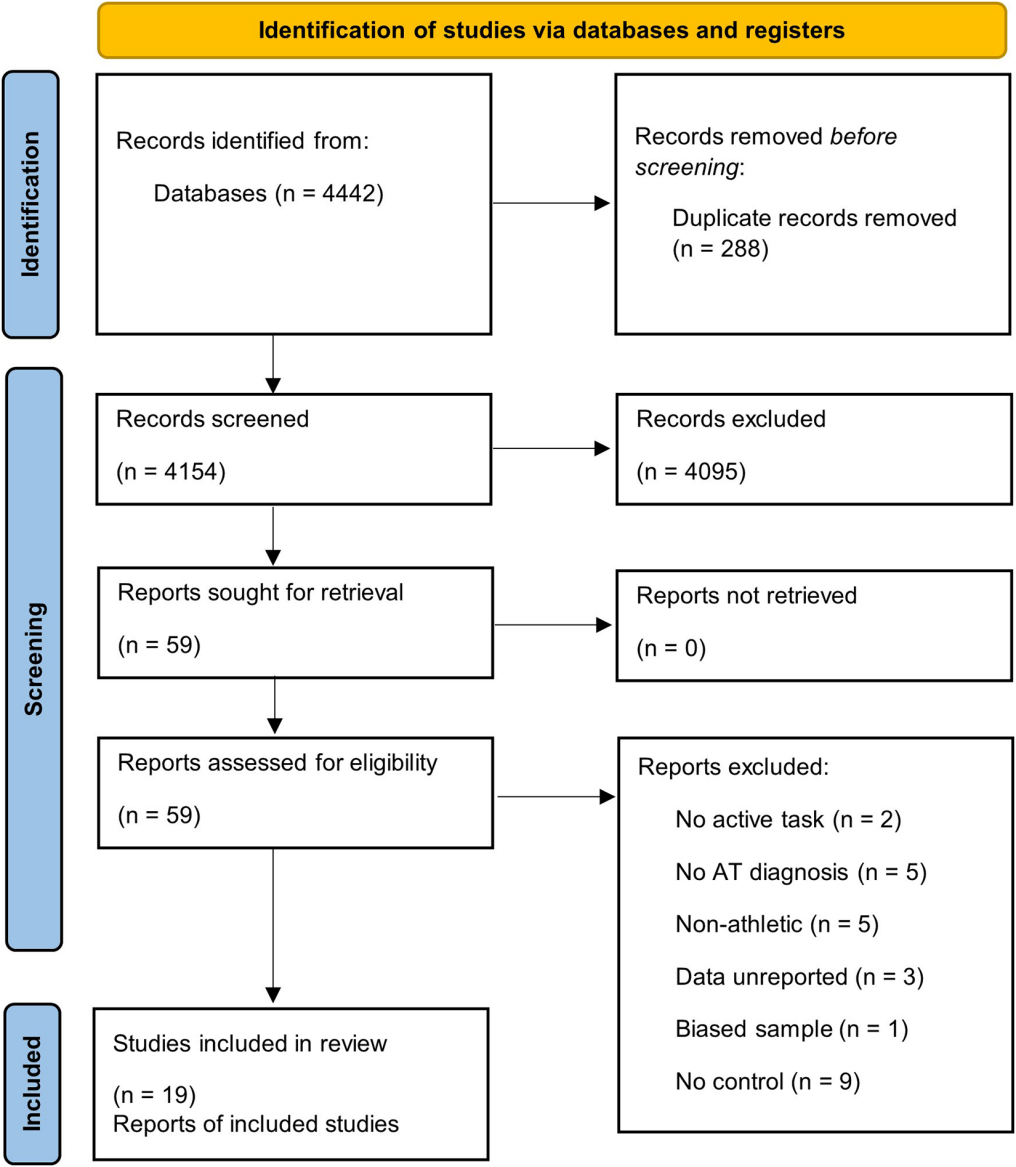
PRISMA flow diagram.

A total of 4,442 studies were yielded with the initial search criteria. After excluding studies *via* title and abstract, 59 full-text studies were identified as potentially suitable. Twenty of these full-text reports (taken from 19 experimental study populations) met the inclusion criteria and were synthesised for data extraction and analysis within this review. Details of this process can be seen in the PRISMA flow diagram (Figure 1). Eighteen of the included studies were case-control study designs, whilst 2 of the studies were prospective (32, 33). Concerning task characteristics, 11 of the studies examined “Gait” characteristics (32–42), 3 studies reported on biomechanical variables during a “Non-gait multi-joint activity” (23, 43, 44), 8 of the studies investigated “Isolated joint strength” (32, 43, 45–50), and only 1 study explored “Reflex activity” (23). Nineteen of the included studies were in the English language, whereas one was written in German (48).

### 3.2. Participants

Details of the included studies can be found in Table 2, with information on participants, task characteristics, relevant outcomes, and calculated effect sizes with 95% confidence intervals. All studies included patients with Achilles tendinopathy (AT) and compared them to a healthy control group. A total of 769 participants were included across all 20 reports (19 experimental study populations). Seven studies included specifically male participants (34, 39–41, 43, 45, 48), a single study investigated females only (44), 9 experiments studied both males and females (32, 33, 36, 37, 42, 46, 47, 49, 50), and three studies did not report the sex of participants (23, 35, 38). The average age of all participants included was 38 years, with a large range from 18.5–50.5 years. Participants in the AT group were three years older on average across all studies (39.2 years vs. 36.2 years). All participants were considered athletic (ranging from recreational to elite), based upon inclusion criteria highlighted within the methodology. The majority of the included studies investigated runners exclusively (*n* = 16) (32, 33, 45–47, 34–40, 42), whereas three studies included running and other sports, such as basketball, soccer, tennis, volleyball, long jump, high jump and ice hockey (23, 43, 49), and a single study examined female dancers only (44). Methods of diagnosis for AT varied substantially, whereby ten studies identified “Achilles tendinopathy” (23, 32–34, 37, 38, 41, 42, 44, 49), five studies diagnosed unilateral “mid-portion Achilles tendinopathy” (35, 36, 43, 48, 50), three studies identified “mid-portion Achilles tendinopathy” without reference to side (39, 40, 45), and two studies included patients with both “insertional and mid-portion Achilles tendinopathy” (46, 47). Twelve of the 20 studies reported AT symptoms duration, and the range of duration was large (>2 weeks–27 months).

**Table 2:** Study characteristics and results.

Authors, Study, Year & Study Type	Sample Group and number (*n*)/male-female/age [years (yr)]/mass (kg), height (cm), BMI (kg/m^2^) Sporting activity [years (yr)]/training per week/diagnosis (duration symptoms)/symptoms during testing	Task characteristics and category	Biomechanical variables	Significant findings (AT vs. CO) (*p* < 0.05) Relative direction of differences shown with arrow for AT group e.g., ↓ = decrease in variable compared to control	*P*-values	Calculated effect sizes (d) + (95% confidence intervals)
Baur et al. (2004) Case control	AT *n* = 8/NR/36 ± 9 yr/73 kg, 179 cm, NR Runners (NR)/5 ± 2 units/unilateral MPAT (>3 months)/symptomatic	'Gait' Run/12 km/h/treadmill/Gymnastic barefoot shoe and standard running shoe	- Antero-posterior and vertical ground reaction forces - Plantar pressure distribution - EMG: Tibialis anterior, peroneus longus, gastrocnemius medialis, gastorcnemius longus and soleus	- Lateral deviation of centre of pressure whilst barefoot ↓	<0.001	−0.89 (−1.79, 0.03)
	CO *n* = 14/NR/36 ± 9 yr/73 kg, 179 cm, NR Runners (NR)/5 ± 2 units			- EMG amplitudes of extensor loop both barefoot and shod in weight acceptance ↓	<0.001	NA
Baur et al. (2011) Case control	AT *n* = 30/19m10f/41 ± 7 yr/72 kg, 175 cm, NR Runners (NR)/45 ± 21 km/unilateral MPAT (>3 months)/symptomatic	'Gait' Run/12 km/h/treadmill Neutral running shoes	- EMG: tibialis anterior, peroneus longus and gastrocnemius medialis (Plantar pressure distribution used for calculation of touch down and take off time)	- Peroneus longus weight acceptance ↓	0.006	−0.54 (−1.05, −0.02)*
	CO *n* = 30/20m10f/37 ± 10 yr/67 kg, 174 cm, NR Runners (NR)/42 ± 14 km			- Gastrocnemius medialis weight acceptance ↓	0.001	−0.63 (−1.14, −0.10)*
				- Gastrocnemius medialis push-off ↓	0.04	−0.40 (−0.91, 0.12)
Becker et al. (2017) Case control	AT *n* = 13/9m4f/37.6 ± 15.9 yr/NR, NR, NR Runners (NR)/50.1 ± 15.1 miles/AT (NR)/symptomatic	'Gait' Run/Self-selected pace/5-m straight section Own running shoes	- 3D foot kinematics	- Rearfoot eversion heel-off ↑	<0.001	−1.63 (−2.51, −0.72)*
	CO *n* = 13/9m4f/32.6 ± 12.4 yr/NR, NR, NR Runners (NR)/52.32 ± 14.7 miles		- Ground reaction forces	- Period of pronation ↑	<0.001	1.72 (0.80, 2.62)*
Bramah et al. (2018) Case control (sub-group analysis)	AT *n* = 18/NR/38.5 ± 11.7 yr/63.1 ± 11.8 kg, 171.6 ± 8.7 cm, 21.3 ± 2.0 kg/m^2^/ Runners (NR)/31.9 ± 17.6 miles/AT (>3 months)/symptomatic (pain not >3 on NRS)	'Gait' Run/3.2 m/s (11.5 km/h)/treadmill Own running shoes	- Kinematics at initial contact and mid-stance of trunk, pelvis, hip, knee and ankle.	- Contralateral pelvic drop midstance ↑	<0.01	1.37 (0.74, 1.99)*
	CO *n* = 36/15m21f/33.2 ± 8.4 yr/60.8 ± 8.4 kg, 171.6 ± 7.3 cm, 20.6 ± 1.8 kg/m^2^ Runners (NR)/60.5 ± 23.2 miles			- Knee flexion initial contact ↓	<0.01	−0.87 (−1.46, −0.28)*
				- Ankle dorsiflexion initial contact ↑ (differences reported for pooled group of 4 separate injuries, but differences were consistent between injuries by sub-group analysis)	<0.01	0.72 (0.14, 1.30)*
Chang et al. (2015) Case control	AT *n* = 9/NR/46.8 ± 6.3 yr/74.1 kg, 170 cm, NR Runners, basketball, soccer, tennis (NR)/4,720 MET-min/AT (>2 wks)/asymptomatic (pain history)	'Non-Gait Multi-joint Activity' Hop/20 submaximal single leg hops/frequency 2.2 Hz/Own shoes	- Hopping: EMG - tibialis anterior, medial gastrocnemius, soleus and peroneal longus (Ground reaction forces for calculation of touch down and take off)	- "Individuals with Achilles tendinosis exhibit an earlier preactivation of the medial gastrocnemius on the involved side."	<0.001	3.42 (1.94, 4.85)*
	CO *n* = 10/NR/48.7 ± 4.4 yr/84.9 kg, 170 cm, NR NR/3,983 MET-min	'Reflex Activity' Electrical stimulation of tibial nerve and evoked muscle activity	- Reflex activity: Electromechanical delay, spinal and supraspinal responses	- "Meanwhile, the lowered contribution to plantar flexion from the triceps surae muscle was compensated for by other plantar flexors, such as the peroneal longus"	<0.001	−2.33 (−3.50, −1.12)*
				- "Both the H-reflex and the V-wave were higher on the side of Achilles tendinosis"	<0.001	H - 1.99 (0.85, 3.09)* V - 1.79 (0.69, 2.85)*
Child et al. (2010) Case control	AT *n* = 14/14m0f/40 ± 8 yr/80 ± 9 kg, 177 cm ± 6 cm, NR Runners (NR)/48 ± 18 km/MPAT (27 months)/symptomatic	'Isolated Joint Strength' Maximal strength: isometric PF contraction with customized calf-raise apparatus with load cell/2-sec ramp followed by 3-sec MVC	- Isometric PF force (N)	- No significant differences between groups AT = 826.5 ± 246.8 N vs. CO = 755.6 ± 214.3 N	0.393	0.30 (−0.43, 1.03)
	CO *n* = 15/15m0f/35 ± 9 yr/79 ± 11 kg, 178 cm ± 5 cm, NR Runners (NR)/42 ± 13 km					
Creaby et al. (2017) Case control	AT *n* = 14/14m0f/43 ± 8 yr/82.3 kg, 179 cm, 25.73 kg/m^2^ Runners (NR)/38.1 ± 13.2 km/MPAT (NR)/Symptomatic	'Gait' Run/4 m/s (±10%)/25 m walkway Shoes: Nike Strap Runner IV	- 3D Kinematics: hip and ankle joints - Kinetics: joint moments of hip and ankle - Isometric plantar flexor peak torque (Nm)	- Hip external rotation at peak vGRF ↓	0.042	0.67 (−0.15, 1.48)
	CO *n* = 11/11m0f/37 ± 9 yr/73.5 kg, 177 cm, 23.5 kg/m^2^ Runners (NR)/35.9 ± 13.6 km			- Hip peak external rotation joint moment ↑	0.0006	1.60 (0.67, 2.50)*
				- Hip external rotation impulse ↑	0.0001	1.50 (0.59, 2.39)*
				- Hip adduction impulse ↑	0.0005	1.67 (0.73, 2.58)*
Crouzier et al. (2020) Case Control	AT *n* = 21/18m3f/36.2 ± 8.3 yr/72.7 ± 8.7 kg, 175.9 cm ± 8.0 cm, 23.4 ± 1.5 kg/m^2^ Mostly runners (NR)/3903 ± 2,105 MET·min·wk (2 h 36 min average of running per week)/insertional (*n* = 5) and MPAT (*n* = 16) (3.6 months); 4 patients with bilateral symptoms/Asymptomatic	'Isolated Joint Strength' Maximal Strength: MVIC of plantar flexors *via* isokinetic dynamometery, knee fully extended. 4 × 3 s.		- No differences in maxmal peak torque	0.403	−0.26 (−0.87, 0.35)
	CO *n* = 21/18m3f/35.1 ± 7.7 yr/71.8 ± 10.5 kg, 177.5 cm ± 7.8 cm, 22.7 ± 2.4 kg/m^2^ Mostly runners (NR)/4601 ± 1,983 MET·min·wk (2 h 36 min average of running per week)	Submaximal strength: Isometric plantar flexor target torques of 20% and 40% of MVIC. 6 × 8 s trials at both intensities.	- Index of individual muscle (distribution of muscle force) %	- "We observed a different force distribution between the two populations with the GL muscle contributing significantly less to the overall submaximal isometric plantarflexion force in people with Achilles tendinopathy compared with the controls."	0.025	−0.54 (−1.15, 0.08)
			- EMG: Soleus, gastrconemius medialis, gastrocnmeius lateralis			
Ferreira et al. (2020) Case control	AT *n* = 25/20m5f/36.84 ± 8.36 yr/73.90 ± 11.65 kg, 171 cm ± 6 cm, 24.97 ± 3.55 kg/m^2^ Runners (6.7 ± 5.5 yrs)/36 ± 16.6 km/ MPAT & IAT mix (NR)/Asymptomatic	'Isolated Joint Strength' Isometric Maximal Strength (MVIC) of plantars flexors and hip external rotators using handheld dynamometer setup. 4 × 5 s MVIC, with 30 s rest.	- Isometric torque (Nm/Kg) for ankle plantarflexors and hip external rotators.	- "Runners with hip IR ROM under 13.99°, ankle PF torque above 0.76 Nm/kg, SFA above 5.53° and hip ER torque above 0.61 Nm/kg have higher chance of having AT."	<0.001	PF: 0.21 (−0.34, 0.76) Hip: −0.28 (−0.83, 0.27)
	CO *n* = 26/21m5f/35.07 ± 9.36 yr/73.55 ± 11.06 kg, 169 cm ± 7 cm, 25.4 ± 2.4 kg/m^2^ Runners (6.2 ± 5.8 yrs)/40.15 ± 21.27 km		- Hip internal rotation ROM and shank-forefoot alignment.	- "with hip IR ROM ≤29.33°, ankle PF torque >0.76 Nm/kg, and SFA >5.53°, hip ER torque was added into the model with a cut-off point of 0.61 Nm/kg. In this case, runners with hip ER torque below this value were identified with AT."		
Franettovich et al. (2014) Case control	AT *n* = 14/14m0f/43 ± 8 yr/82.3 kg, 179 cm, NR Runners (NR)/38.1 ± 13.2 km/MPAT (NR)/Symptomatic	'Gait' Run/4 m/s (±10%)/25 m walkway Shoes: Nike Strap Runner IV	- EMG: Gluteus medius and gluteus maximus (vertical ground reaction forces used to identify heel strike and toe-off)	- Gluteus medius onset delay	<0.001	2.11 (1.23, 2.96)*
	CO *n* = 19/19m0f/37 ± 8 yr/77.4 kg, 179 cm, NR Runners (NR)/37.6 ± 16.4 km			- Gluteus medius activity duration ↓	<0.001	2.31 (1.40, 3.19)*
				- Gluteus maximus onset delay	0.008	1.41 (0.62, 2.17)*
				- Gluteus maximus activity duration ↓	0.002	1.82 (0.98, 2.63)*
				- Gluteus maximus offset early	0.001	1.50 (0.71, 2.27)*
Habets et al. (2016) Case control	AT *n* = 12/12m0f/51.5 (43.0–53.0) yr/80.5 (75.0–92.9)kg, 189.0 (181.3–192.0)cm, 22.7 (21.8–26.8)kg/m^2^ Runners, soccer, volleyball and tennis (34 yrs)/<3 h–>7 h/unilateral MPAT (17.5 months)/NR	'Isolated Joint Strength' Isometric maximal strength (MVIC) of hip abductors, hip external rotators and hip extensors, measured with HHD.	- Isometric strength (N/KgBw): Hip abductors, hip external rotators and hip extensors.	- Hip abductor strength ↓	0.012	NA
	CO *n* = 12/12m0f/49.5 (42.0–53.5) yr/80.5 (69.5–88.9)kg, 181.5 (179.3–185.8)cm, 23.7 (21.7–26.2)kg/m^2^ Runners, soccer, volleyball and tennis (40 yrs)/<3 h–>7 h	'Non-Gait Multi-joint Activity' Functional hip performance - single leg squat (barefoot and underwear)	- Subjective rating of single-leg squat, based on "movement quality" criteria.	- Hip external rotator strength ↓	0.010	
				- Hip extension strength ↓	0.034	
				- No significant difference in functional hip performance		
Hein et al. (2013) Prospective	AT *n* = 10/8m2f/45 ± 5 yr/72 kg, 177 cm, 23 kg/m^2^ Runners (NR)/33 ± 15 km/AT/ asymptomatic (healthy participants)	'Gait' Run/12 km/h/13 m runway Barefoot 'Isolated Joint Strength' Isometric strength: Hip abduction/adduction and knee flexion/extension	- Kinematics of hip, knee and ankle joints	- "As maximal joint excursions correlate with initial joint angles, it can be concluded that AT also show a more extended knee joint, a lower dorsiflexed ankle joint and a more everted rearfoot at touchdown compared with CO."	NR	Knee: −0.58 (−1.47, 0.33) DF: −1.21 (−2.16, −0.24)* EVR: −0.57 (−1.45, 0.34)
	CO *n* = 10/8m2f/40 ± 7 yr/72 kg, 177 cm, 23 kg/m^2^ Runners (NR)/32 ± 20 km		- Maximal torque (Nm)	- "Runners who developed AT already showed decreased knee flexor strength compared with CO in an uninjured state even though 95% confidence intervals slightly overlap. No differences in maximal isometric strength were found for the hip joint surrounding muscles, or knee extensors between AT and CO."	NR	−0.91 (−1.83, 0.02)
Hirschmüller et al. (2005) Case control	AT *n* = 72/72m0f/39.4 ± 6.3 yr/74.0 ± 7.9 kg, 178.2 ± 5.7 cm, NR Runners (13.8 ± 8.7)/49.6 ± 5, 2 km/unilateral MPAT (NR)/NR	'Isolated Joint Strength' Maximum concentric and eccentric strength of ankle plantar flexion and dorsi flexion, isokinetic dynamometry.	- Maximum concentric and eccentric torque of ankle plantar flexion and dorsi flexion, measured with isokinetic dynamometry (velocity: 60°/s).	- Plantar flexor torque (Nm) both concentric and eccentric ↓	<0.05	NA
	CO *n* = 20/20m0f/28.7 ± 7, 9 yr/72.7 ± 9.6 kg, 180.6 cm ± 6.1 cm, NR Runners (8.2 ± 4.6)/37.0 ± 12.7		- EMG: Tibialis anterior, gastrocnemius medialis, gastrocnemius lateralis and soleus.	- EMG amplitudes gastrocnemius medialis, gastrocnemius lateralis and soleus during PF ↑	<0.01	
			- Neuromuscular efficiency quotient	- Neuromuscular efficiency ↓	<0.01	
Kulig et al. (2011) Case Control	AT *n* = 8/0m8f/18.5 ± 1.1yr/57.3 kg, 164 cm, NR Dancing (NR)/30 to 35 h/AT (>3 months)/asymptomatic (history of pain)	'Non-Gait Multi-joint Activity' Hop/saut de chat jump (maximal effort/height jump common in ballet) - minimum 3 trials Without shoes	- Kinematics: Hip, knee and ankle joints.	- Hip adduction during braking phase ↑	0.046	1.04 (−0.03, 2.07)
	CO *n* = 8/0m8f/19.0 ± 1.3 yr/54.3 kg, 164 cm, NR Dancing (NR)/30 to 35 h			- Knee internal rotation during push-off phase ↑	0.024	1.25 (0.15, 2.31)*
Masood et al. (2014) Case Control	AT *n* = 11/7m4f/28 ± 4 yr/66 ± 6 kg, 174 cm ± 6 cm, NR Running, long jump, high jump, ice hockey (NR)/4.7 units/AT (9.8 months)/Asymptomatic	'Isolated Joint Strength' Maximal plantar flexor strength: isometric contraction *via* an in-house custom-built portable force transducer MVIC 8 sets of 5 unilateral, isometric, submaximal contractions at 30% of MVIC.	- MVIC PF force, N 30% MVIC, N	- "Normalized myoelectric activity of soleus was higher (*P* < 0.05) in the symptomatic leg vs. the contralateral and control legs despite lower absolute force level maintained (*P* < 0.005).	<0.005	1.4* (NA)
	CO *n* = 11/7m4f/28 ± 4yr/67 ± 6 kg, 173 cm ± 6 cm, NR Running, long jump, high jump, ice hockey (NR)/2.4 units		- EMG: Soleus, gastrocnemius medialis, gastrocnemius lateralis and hallucis longus	- No difference for maximal PF force between AT and CO		
O'Neill et al. (2019) Case control	AT *n* = 39/34m5f/47 ± 11.8yr/77 ± 12.1 kg, 177 cm ± 6.8 cm, 24 ± 2.7 kg/m^2^/ Endurance runners (NR)/15–30 miles/unilateral MPAT (>3 months)/Asymptomatic	'Isolated Joint Strength' Maximal plantar flexor strength: measured by isokinetic dynamometry. Tested knee full extension and knee flexion 80° – concentric 90°/sec, concentric 225°/sec and eccentric 90°/sec.	- Peak plantar flexor torque (Nm & % body weight)	- "The results clearly show that there are large deficits in strength between subjects with and without AT. The magnitude of deficits is clinically and statistically significant in all test modes and both knee positions."	<0.001	Range: −0.83 (−1.30, −0.36*, −1.79 (−2.31, −1.25)*
	CO *n* = 38/35m3f/44 ± 9.9yr/70.4 ± 10.3 kg, 175 cm ± 8.1 cm, 23 ± 2.7 kg/m^2^/ Endurance runners (NR)/15–30 miles	Plantar flexor endurance: 20 maximal effort concentric-eccentric plantar flexor contractions, positioned knee flexion 80°.	- Endurance capcity plantar flexors (Total Work Done)	- "The small percentage difference in force (healthy control torque/AT torque) observed between knee flexion and extension suggests that the gastrocnemius accounts for between 3.7%–11% of the identified deficits, whilst the soleus may be responsible for the remaining 23.2%–36.1% of the difference"		−1.25 (−1.73, −0.76)*
				- "The endurance data shows a clear clinically meaningful and statistically significant difference between subjects with and without AT"		
Ryan et al. (2009) Case control	AT *n* = 27/27m0f/40 ± 7yr/78 kg, 181 cm, NR Runners (>6 months)/>30 km/ AT (27 months)/symptomatic	'Gait' Run/pace self-selected/15 m runway Without shoes	- Kinematics: Ankle (frontal and sagittal plane), tibia (transverse plane)	- Sub-talar joint eversion (mid-stance) ↑	0.04	0.67 (0.08, 1.25)*
	CO *n* = 21/21m0f/40 ± 9yr/71 kg, 177 cm, NR Runners (>6 months)/>30 km			- No difference in transverse tibial motion		
Van Ginckel et al. (2009) Prospective	AT *n* = 10/2m8f/38 ± 11.35yr/69.8 kg, 167,1 cm, 24.95 kg/m^2^ Runners (Novice)/7.7 ± 1.03 h (walk + run)/AT/asymptomatic (healthy participants)	'Gait' Run/Self-selected speed/15 m runway Barefoot	- Plantar pressure force distribution	- Total posterior–anterior displacement of the Centre Of Force ↓	0.015	−0.95 (−1.65, −0.25)*
	CO *n* = 53/8m45f/40 ± 9yr/69.95 kg, 168.34 cm, 24.69 kg/m^2^ Runners (Novice)/7.8 ± 1.24 h (walk + run)			- Laterally directed force distribution underneath the forefoot at “forefoot flat” ↑	0.016	−0.93 (−1.62, −0.23)*
Williams et al. (2008) Case control	AT *n* = 8/6m2f/36.0 ± 8.2yr/67.3 kg, 176 cm, NR Runners (19.1 ± 7.7 yrs)/41.3 ± 20.8 km/AT/Asymptomatic (pain history)	'Gait' Run/3.35 m/s (± 5%)/20 m runway Saucony shoes/rear foot strikers	- Kinematics and joint moments in transverse plane of knee and tibia	- Tibial external rotation moment stance ↓	0.01	1.36 (0.24, 2.44)*
	CO *n* = 8/5m3f/31.8 ± 9.3yr/65.6 kg, 170 cm, NR Runners (11 ± 9.1 yrs)/35.3 ± 23.1 km			- Peak knee internal rotation stance ↓	0.05	−0.97 (−2.00, 0.09)
Wyndow et al. (2013) Case Control	AT *n* = 15/15 m0f/42 ± 7yr/80 kg, 177 cm, NR Runners (NR)/>20 km; maximum of 43 ± 15 km/AT (>3 months)/symptomatic	'Gait' Run/4 m/s/25 m runway Shoes: Nike strap runners	- EMG: Soleus, gastrocnemius lateralis, gastrocnemius medialis (Ground reaction forces utilised to identify heel strike and toe-off)	- Earlier offset of soleus EMG activation relative to gastrocnemius lateralis	0.02	−0.90 (−1.60, −0.18)
	CO *n* = 19/19m0f/36 ± 8yr/77 kg, 179 cm, NR Runners (NR)/>20 km; maximum of 40 ± 16 km					

* Statistical significance; NR, not reported; NA, not available; AT, achilles tendinopathy/participant with achilles tendinopathy; CO, control group; MPAT, mid-portion achilles tendinopathy; NRS, numerical rating scale; MET, metabolic equivalents; IAT, insertional achilles tendinopathy; Km, kilometres; Cm, centimetres; m, metre; m, male; f, female; BMI, body mass index; Kg, kilograms; Hrs, hours; Mins, minutes; Wk, week; Km/h, kilometres per hour; Secs, seconds; PF, plantar flexion; MVC, maximum voluntary contraction; MVIC, maximum voluntary isometric contraction; HHD, handheld dynamometry; EMG, electromyography; Hz, hertz; 3D, three dimensional; Nm, Newton metres; N, Newtons; ROM, range of motion; vGRF, vertical ground reaction force; IR, internal rotation; ER, external rotation; SFA, Shank-forefoot alignment; DF, dorsiflexion; EVR, eversion.

### 3.3. Outcomes

#### 3.3.1. Gait activities

All 11 studies examining gait (32, 33, 42, 34–41) investigated running, at a variety of speeds and under different shod/barefoot conditions (see Table 2). None of the included studies researched other forms of human gait e.g., walking.

##### 3.3.1.1. Kinematics

One prospective study investigated the kinematics of the hip, knee and ankle joints in twenty participants during running (32), and concluded that a more extended knee joint, a decreased angle of dorsiflexion at the ankle joint and a more everted rearfoot at touchdown preceded onset of AT. The remaining five studies investigated kinematics cross-sectionally. Four studies investigated ankle kinematics during running (37–39, 41). One study (37) reported changes in ankle kinematics with AT, including increased rearfoot eversion at heel-off and an increased period of pronation. Another study (41) also showed increased sub-talar joint eversion at mid-stance but no differences in ankle sagittal plane kinematics. One study (38) reported that AT patients exhibit increased ankle dorsiflexion at initial contact, but no differences in rearfoot frontal plane kinematics. Another study (39) showed no changes in sagittal nor frontal plane ankle kinematics for AT patients compared to healthy controls. One study (41) reported no difference in transverse tibial motion. A single study (38) reported reduced knee flexion at initial contact in the AT group, but no changes in knee kinematics in either the sagittal, transverse or frontal planes during midstance. A different study (42) showed a decrease in peak knee internal rotation angles within the AT group. One study (38) reported increased contra-lateral pelvic hip drop in the AT group compared to controls. A separate study (39) showed a difference in hip kinematics, namely a reduction in hip external rotation at peak ground reaction force (GRF), but not for four other variables in the sagittal and frontal planes.

##### 3.3.1.2. Joint moments

Only two included studies investigated joint moments during running (39, 42). One study (39) reported no differences in ankle joint moments between groups, but demonstrated a decreased hip peak external rotation joint moment, hip external rotation impulse and hip adduction impulse in the AT group, with no differences in the sagittal plane. Another study (42) indicated a decreased tibial external rotation moment during stance phase, with no differences in transverse plane knee joint moment.

##### 3.3.1.3. Ground reaction force

Two studies (35, 37) reported on GRF during running. Neither of these studies indicated any differences in vertical or propulsive and braking GRFs between the AT and a healthy control group.

##### 3.3.1.4. Plantar pressure force distribution

A single prospective study (33) reported a significant decrease in posterior–anterior displacement of the centre of force and a laterally directed force distribution underneath the forefoot at “forefoot flat” during running, indicating AT onset in a prospective study design. Another study investigated plantar pressure force distribution cross-sectionally during running (35), showing a decreased lateral deviation of the centre of pressure in relation to the midline of the foot in AT whilst running barefoot.

##### 3.3.1.5. Muscle activity (EMG)

A total of four studies investigated electromyographic changes (EMG) during running (34–36, 40). *Gastrocnemius:* One study (35) reported decreased amplitudes of the gastrocnemius lateralis during weight acceptance in the AT group, with no differences reported in timing. Another study (36) indicated reduced amplitudes of the gastrocnemius medialis during weight acceptance and push-off. A separate study (34) showed reduced offset EMG timing of the soleus relative to lateral gastrocnemius, although five other variables relating to muscle activity timing of the calf complex were not statistically significant. *Soleus:* One study (34) reported earlier offset of the soleus relative to gastrocnemius, whereas another study did not report any differences between groups (35). *Peroneus longus:* Two studies (35, 36) reported no differences in peroneus longus activity during pre-activation, although one of these studies (36) did show decreased activity during weight acceptance within the AT group. *Tibialis anterior:* Both studies (35, 36) reported no differences in tibialis anterior EMG activity between the AT group and controls. *Hip muscles:* One study (40) investigated EMG in muscles of the hip and reported a delayed gluteus medius onset, reduced gluteus medius activity duration, delayed onset of gluteus maximus, reduced gluteus maximus activity duration and earlier offset of gluteus maximus in the AT group.

#### 3.3.2. Non-gait multi-joint activities

##### 3.3.2.1. Sub-maximal hopping

One study (23) investigated hopping, finding that the AT group had a relatively lower contribution of the gastrocnemius and soleus muscles, compensated for by increased peroneus longus activity as measured by EMG.

##### 3.3.2.2. Maximal jump

A single study (44) investigated the “saut de chat” ballet jump in dancers, and reported increased hip adduction during braking phase and increased knee internal rotation during push-off phase in the AT group, as measured by 3D kinematics of the hip, knee and ankle.

##### 3.3.2.3. Functional hip performance

One study (43) subjectively assessed the function of the hip based upon pre-defined “movement quality” criteria during a single-leg squat, and reported no differences between the AT and control group in the subjective visual rating of postural stability and movement execution. The rating was based upon movement quality criteria in five domains and was subjectively rated by the investigators *via* video analysis in post-processing, whereby ratings for the domains were categorised as “poor”, “fair”, or “good” and then indexed into a total score.

#### 3.3.3. Isolated joint strength

Eight of the studies investigated “Isolated joint strength” (32, 43, 45–50), and reported on a wide range of biomechanical strength variables. Measurement techniques varied, including isokinetic dynamometry (46, 48, 50), handheld dynamometry (43, 47) and other custom made devices (32, 45, 49). Subject positioning also differed between studies to a large degree, depending on apparatus used and the joint of interest. Six studies reported on strength of the ankle joint (45–50), one study investigated the knee joint (32) and three studies reported on the hip joint (32, 43, 47).

##### 3.3.3.1. Maximal strength

Only one study of twenty subjects (32) investigated maximal isometric strength prospectively, and identified decreased knee flexor strength in runners who went on to develop AT. No differences in maximal isometric strength were found for the hip joint surrounding muscles, or knee extensors between AT and control subjects. Regarding cross-sectional study designs, a total of six studies investigated maximal strength of the ankle joint (45–50). Two studies (48, 50) found associations between reduced maximal plantar flexor (PF) strength in the AT group, during both concentric and eccentric muscle contractions on an isokinetic dynamometer. In one of these studies (50), the effort was produced with the knee both fully extended and bent at 80°. One other study (47) reported that increased isometric PF strength was associated with AT, but only when associated with other biomechanical factors. Three different studies (45, 46, 49) discovered no differences in isometric PF strength between the AT group and heathy controls. One study (43) investigated isometric maximal strength of the hip, and reported reduced hip abductor strength, reduced hip external rotator strength and decreased hip extension strength in the AT group. A separate study (47) reported that both increased and decreased isometric hip external rotation strength when combined with other biomechanical factors, were associated with AT.

##### 3.3.3.2. Strength endurance

One study (50) investigated plantar flexor endurance (20 repetition protocol) *via* isokinetic dynamometry, and reported significant and clinically meaningful deficits in the AT group compared to healthy controls.

##### 3.3.3.3. Muscle activity (EMG)

A total of three studies (46, 48, 49) investigated muscle activity during isolated joint strength activities. All studies measured strength of the ankle joint in plantar flexion, whilst one study also measured dorsi flexion (48). One study (46) measured soleus, gastrocnemius medialis and gastrocnemius lateralis EMG activation, and reported a lower contribution of gastrocnemius lateralis activity to overall triceps surae output in the AT group, during sub-maximal intensities [20% and 40% of maximum voluntary isometric contraction (MVIC)]. Two studies showed increased EMG activity of the soleus (49), and soleus, gastrocnemius medialis and gastrocnemius lateralis muscles (48) within AT patients, despite lower levels of overall plantar flexor force output in both studies.

#### 3.3.4. Reflex activity

Only a single study (23) investigated reflex activity. The authors reported an up-regulated spinal reflex at rest (H-reflex) and accentuated supraspinal reflex responses (V-Wave) during MVIC, on the involved side of AT patients when compared to healthy controls.

### 3.4. Risk of bias

The risk of bias assessment according to the Critical Skills Appraisal Checklist can be seen in Table 3. Overall, it could be concluded that the included studies scored poorly in “Appropriate Recruitment of Controls”, “Control of Confounding Factors” and “Generalizability of the Results”. There was a wide variation of methodological approaches applied, especially regarding recruitment of controls, symptoms of injury, footwear, positioning of the participants, the measurement techniques utilised and the statistical designs of the studies. Therefore, it could be concluded that risk of bias in the included studies was predominantly moderate/high.

**Table 3:** Risk of bias assessment for included studies.

Study and year	Focused Issue?	Appropriate Methodology?	Apprpriate Recruitment of Patients?	Appropriate Recruitment of Controls?	Exposure Accurately Measured?	Control of Confounding Factors?	Precision of Results?	Statistics Clearly Reported?	Generalizability of Results?	Results Fit With Other Evidence?
Baur et al. (2004)	Yes	No	No	No	Yes	No	Yes	Yes	No	Yes
Baur et al. (2011)	Yes	Yes	Yes	No	Yes	No	No	Yes	No	Yes
Becker et al. (2017)	No	Yes	No	No	No	No	Yes	No	No	Yes
Bramah et al. (2018)	No	Yes	Yes	Yes	Yes	No	No	No	Yes	Yes
Chang et al. (2015)	Yes	Yes	Yes	Yes	Yes	No	No	Yes	No	Yes
Child et al. (2010)	Yes	Yes	No	No	Yes	No	Yes	Yes	Yes	Yes
Creaby et al. (2017)	Yes	Yes	Yes	No	Yes	No	Yes	Yes	No	No
Crouzier et al. (2020)	Yes	Yes	Yes	No	Yes	No	Yes	No	No	Yes
Ferreira et al. (2020)	No	No	No	No	Yes	No	No	Yes	No	No
Franettovich et al. (2014)	Yes	Yes	Yes	No	Yes	No	Yes	Yes	No	Yes
Habets et al. (2016)	Yes	No	Yes	No	No	No	No	Yes	No	Yes
Hein et al. (2013)	Yes	Yes	No	No	Yes	No	No	No	No	Yes
Hirschmüller et al. (2005)	Yes	Yes	Yes	No	Yes	No	Yes	Yes	No	Yes
Kulig et al. (2011)	Yes	No	Yes	Yes	Yes	No	No	No	No	Yes
Masood et al. (2014)	Yes	Yes	Yes	No	No	Yes	No	No	No	No
O'Neill et al. (2019)	Yes	Yes	Yes	No	Yes	Yes	No	Yes	No	Yes
Ryan et al. (2009)	Yes	No	Yes	Yes	Yes	No	Yes	No	No	Yes
Van Ginckel et al. (2009)	Yes	Yes	Yes	Yes	Yes	No	Yes	No	No	No
Williams et al. (2008)	Yes	Yes	Yes	No	Yes	No	No	No	Yes	Yes
Wyndow et al. (2013)	Yes	No	Yes	No	Yes	No	Yes	No	No	Yes

## 4. Discussion

This study aimed to synthesise the evidence regarding biomechanical alterations and changes in lower limb function in patients with AT. The included studies investigated exclusively athletic populations, from recreational to elite level, when compared to a healthy athletically matched control group. Throughout the discussion, effect sizes and statistical significance are reported from the relevant studies, to allow for clear comparison and interpretation (reported as: Effect Size (ES) d [95% Confidence Intervals (CIs)]. Statistical significance is indicated by an asterisk*). Overall, it must be emphasised that most of the reported biomechanical variables produced conflicting results within this review, especially regarding the data during running and jumping activities. Whilst a number of biomechanical theories have been postulated elsewhere in the literature, for example “medial collapse theory” and “contralateral pelvic hip drop” (20, 38), the authors of the current study do not believe that there is sufficient evidence to support or refute any such theories based upon the research summarised within this review. We would explicitly recommend against drawing concrete conclusions and applying them on absolute terms in clinical practice, until more evidence has been gathered and the picture is clearer. Nonetheless, readers will find an attempt to interpret and discuss the data collected in this review, in the context of popular theories within the realm of sports biomechanics. This should in no way be considered as an endorsement of these theories or approaches.

### 4.1. Potential biomechanical alterations during gait

There is some evidence to suggest that ankle biomechanics may be altered in athletic AT patients, although the results were conflicting, and several variables were not associated with AT. Increased ankle eversion during running was correlated with injury both prospectively [*d* = − 0.57 (−1.45, 0.34)] and cross-sectionally (37, 41) (*d* = − 1.63 [−2.51, −0.72]*; *d* = 0.67 [0.08, 1.25]*). An increased period of pronation during running was also found to identify patients with AT (37), with strong ES [*d* = 1.72 (0.80, 2.62)*]. This data potentially corroborates previous suggestions that over-pronation of the foot may produce a “whiplash effect” (51), placing excessive strain on the Achilles tendon and leading to injury. This theory is further supported by evidence of increased medial deviation of the foot whilst running, as measured by plantar pressure distribution (35) [*d* = − 0.89 (−1.79, 0.03)]. However, in two of these studies, 95% CIs of the ES overlapped zero (32, 35), indicating less statistically robust results. The crossing of the zero was nevertheless particularly small in one study [0.03 (32)], so this should be taken into account. It must also be considered that many studies indicated a large variation in effect sizes, perhaps signaling that these factors might be more relevant for some individuals than others, within the various groups studied. Furthermore, two other studies (38, 39) were not able to detect a difference between groups in transverse or frontal plane kinematics at the ankle. Additionally, a prospective study (33) reported an increase in the laterally directed force distribution at forefoot flat phase prior to the onset of AT in novice runners [*d* = − 0.93 (−1.62, −0.23)*], contradicting the proposed over-pronation hypothesis. Besides, a closer look at absolute values of two studies (32, 41) reporting statistically significant differences in rearfoot eversion, reveals mean differences between the AT and control group of 2 degrees ankle eversion. Whether such changes are clinically detectable and/or meaningful, is a question requiring more attention and research. Perhaps these disparities are more representative of natural movement variability, as opposed to true biomechanical differences on reductionist terms (52, 53). Additionally, three of the included studies investigating ankle biomechanics had participants run shod (37–39), whereas the other three studies instructed subjects to run barefoot (32, 33, 41), and such a methodological discrepancy is likely to have influenced outcomes, especially in kinematics of the ankle joint. Finally, studies that only report on plantar pressure distributions during running (33) offer limited value, as the overall kinematic picture of foot loading is absent, and future research should aim to integrate both kinetic and kinematic measurements simultaneously.

 Two studies reported no differences in sagittal plane ankle kinematics when comparing AT patients to healthy controls (39, 41). However, changes in sagittal plane ankle kinematics were reported in two other studies during running (32, 38), with one study showing increased dorsiflexion in the AT group (38) [*d* = 0.72 (0.14, 1.30)*], and a single prospective study associating decreased ankle dorsiflexion with onset of AT (32) [*d* = − 1.21 (−2.16, −0.24)*], whereby ES are moderate to large in either direction. It could be speculated that a more compliant strategy at the ankle as seen in one study (38), meaning increased dorsiflexion range of movement (ROM), may result in higher loads on the Achilles tendon during running and potentially lead to injury (54, 55). However, it seems very difficult to support this hypothesis based upon current evidence, especially in light of findings suggesting that static dorsiflexion range of motion (ROM) might be reduced in AT (37), alongside prospective evidence associating decreased ankle dorsiflexion with onset of AT (32). Perhaps it could be interpreted that both increased and decreased dorsiflexion ROM might be associated with AT, depending upon individual factors. However, it could just be attributed to natural variability, and much more research is required before any conclusions can be made.

Three studies reported on electromyographic outcomes of the ankle muscles during running. Two of these studies showed decreased EMG amplitudes of the plantar flexor muscles during weight acceptance (35, 36) [*d* = − 0.63 (−1.14, −0.10)*], whilst a single study indicated reduced activity of the gastrocnemius medialis during push-off phase (36), though with a small effect size and non-statistically significant 95%CIs [*d* = − 0.40 (−0.91, 0.12)], although the zero was only crossed by a minimal degree (0.12). This may represent a diminished capacity in AT patients of the triceps surae and Achilles tendon unit to attenuate loads eccentrically, and to store and release energy efficiently during propulsion, as seen in healthy running (56). It could also be indicative of inhibitory processes due to the pain often associated with AT, which has been demonstrated to alter motor behavior (27, 57, 58). Additionally, another study (34) indicated altered temporal activation of the triceps surae muscles in patients with AT [*d* = − 0.90 (−1.60, −0.18)*], providing further evidence of potentially compensatory adaptations to persistent injury (25, 57, 58). However, the data from only three studies is not sufficient to draw concrete conclusions, and further research is required.

The data reporting on variables of the hip during gait provides a conflicting picture, and it is very difficult to infer a coherent pattern. There is evidence from one study (38) to suggest that increased contra-lateral pelvic hip drop during running is associated with AT, with a large effect size and robust confidence intervals [*d* = 1.37 (0.74, 1.99)*]. This contrasts with other prospective research (32) highlighting no differences in kinematics of the hip prior to onset of AT. Another study (39) showed decreased hip external rotation ROM at peak vertical ground reaction force in AT, but 95%CIs crossed the value of zero effect [*d* = 0.67 (−0.15, 1.48)] which weakens the findings, although it was by a small degree (−0.15). The same study (39) additionally reported alterations in mechanics of the hip, reporting increased external rotation impulse and joint moments, and increased hip adduction impulse (large ES, see Table 2). In addition, research investigating EMG during running (40) indicates reduced duration and delayed onset of muscle activity in the gluteus maximus and gluteus medius (large ES, especially for gluteus medius, see Table 2) within AT patients compared to controls. Whether these proximal changes occur as a consequence of alterations in local ankle biomechanics relating to AT pathology, or are an isolated feature, is difficult to deduce based upon current evidence. Besides, the data is conflicting and only based upon four studies. It seems plausible that the potential adaptations associated with AT overuse injury throughout the kinetic chain are interrelated (20, 56), though the exact mechanism remains unknown. Alterations in hip biomechanics have been reported in other common running injuries (59), and there is emerging evidence to suggest that interventions targeting gait-retraining e.g., to alter proximal hip kinematics, paired with strengthening interventions, may have a beneficial effect on pain and function (14, 59). Whether such interventions are of true clinical benefit to AT patients requires further investigation. Moreover, most of the included studies were cross-sectional by design. Therefore, whether the above-mentioned biomechanical changes during running are true risk factors that occur prior to AT onset or are adaptations to the condition post-onset, is a question that remains elusive to answer.

### 4.2. Potential biomechanical alterations during non-gait functional activities

Only three studies investigated the biomechanics of AT patients in non-gait functional activities (23, 43, 44), which makes it challenging to draw overall conclusions. One study (23) detected strong effects of a lower contribution of the triceps surae muscles in AT during 20 sub-maximal hops, compensated for by increased peroneus longus activity [*d* = − 2.33 (−3.50, −1.12)*]. These neuromuscular alterations agree with the evidence discussed for running studies (35, 36, 60), and perhaps represent a broad trend, whereby athletic patients with AT present with altered activation of the triceps surae muscles during stretch shortening activities such as running and hopping. Another study (44) found changes in hip and ankle kinematics in female dancers during a “saut de chat” single unilateral maximum jump (jump common in ballet). Effect size for increased hip adduction and increased knee internal rotation were strong but 95%CIs indicate a high level of variability amongst participants (*d* = 1.04 [−0.03, 2.07]; *d* = 1.25 [0.15, 2.31]*). These findings seem to confer with other results in this review (32, 35, 37–40) potentially indicating an overall biomechanical picture of “medial collapse” during dynamic loading of the lower-limb, featuring contralateral pelvic hip drop and increased hip adduction, knee valgus and increased internal rotation, ankle over-pronation and reduced capacity of the hip stabilisers, which may predispose people and/or be associated with the development of AT or other running injuries (61). This neuromechanical pattern is thought to be a particular risk factor in females, and this population might benefit most from interventions targeting these specific motor behaviours (14, 61). However, it must be stressed that the data is conflicting and at times contradictory. In fact, prospective evidence from one study reported lateral foot deviation as a risk factor for AT development (33), which certainly challenges the commonly purported “medial collapse” hypothesis. The evidence presented in this review is not strong enough to be conclusive and should direct future high quality research studies to replicate or reject the findings. Until then, our assumptions are merely based upon speculation. It should also be emphasised that several studies investigated these biomechanical variables and reported no differences between groups. A single study assessed a one-leg squat in AT compared to controls (43), and found no differences in functional hip performance according to standardised subjective criteria. It could be hypothesized that a single-leg squat does not demand stretch shortening activity of the Achilles tendon and kinetic chain of the lower limb, as opposed to running and hopping activities which are known to load the Achilles to a large degree and potentially lead to pathology (2, 8, 9). Although speculative, this might explain why no kinematic differences were found between the groups and perhaps represents a specific kinematic adaptation of AT patients when performing SSC movements, but not during closed chain squatting.

### 4.3. Reduced knee flexor strength prospectively

A single study (32) showed that reduced isometric knee flexor strength predicted onset of AT prospectively, with strong effects but wide 95%CIs [*d* = − 0.91 (−1.83, 0.02)]. The zero is crossed in this instance, but only by a very small margin (0.02) and therefore, is potentially irrelevant. The importance of the hamstring muscles in load attenuation and propulsive sprint efforts is well documented in literature (62) and could form a potential treatment target for AT patients. However, data from a single study is not currently sufficient to make explicit recommendations.

### 4.4. Reduced maximal dynamic plantar flexor strength but not isometrically

Two studies investigated plantar flexor strength dynamically *via* isokinetic dynamometry (48, 50), with both studies showing reduced maximum concentric [*d* = − 1.25 (−1.74, −0.76)*] (50), and eccentric torque [*d* = − 1.38 (−1.88, −0.88)*]. Effect sizes could not be calculated for one of these studies (48) but strength deficits in the AT group were reported as between 10% and 20%, which is less than the 30%–40% deficits seen in absolute values in the comparative study (50). This difference might be explained by the older population included within the O'Neill et al. study (50), which could be correlated with longer duration of symptoms and therefore, exacerbated mechanical adaptations to prolonged pathology. When maximal strength values were normalised to body weight (kg) in one study (50) discrepancies became even more prominent for AT vs. control group, with deficits of 40%–45% reported for the extended and flexed knee positions, in both concentric and eccentric modes and showing strong effect sizes [Max. value: *d* = − 1.79 (−2.31, −1.25)*]. This approach is an interesting avenue for future research and could be easily applied in a clinical setting when working with AT patients. The authors of this study (50) additionally postulate that the gastrocnemius muscle accounts for 3.7%–11% of deficits, whereas the soleus might account for between 23.2%–36.7% of plantar flexor strength deficits. This could specifically imply training of the soleus muscle as a rehabilitation strategy for AT patients, although whether a training intervention is able to specifically target the soleus is still debatable (63, 64). The O‘Neill study (50) also identified significant and clinically meaningful deficits in muscular endurance of the plantar flexors of AT runners, which may be particularly relevant for populations involved in endurance sports requiring repeated loading of the tissues over long periods where fatigue is a factor. Interestingly, three of the four studies investigating isometric plantar flexor strength did not identify any differences between AT and healthy control groups (45, 46, 49). This suggests that isometric testing might not be sufficient to identify strength deficits within an active, athletic population, perhaps alluding to a specific adaptation of the musculotendon unit in AT pathology that does not affect maximal isometric force output. Therefore, despite existing evidence that isometric contractions may have a positive effect on pain and function in tendinopathies (58, 65), isotonic exercises should be considered for strength testing and rehabilitation as soon as symptoms allow. An additional study (47) reported that increased isometric plantar flexor strength was associated with AT, but only in combination with a number of other biomechanical factors when integrated within an interactive statistical model. A closer investigation of absolute values reveals only a 0.05 [Nm normalised to body weight (kg)] difference between groups and small ES with non-statistically significant 95%CIs [*d* = 0.21 (−0.34, 0.76)], bringing into question the clinical relevance of the results.

### 4.5. Alterations in triceps surae activity during plantar flexion

Three studies reported on muscle activity of the triceps surae muscles during isolated ankle plantar flexor strength in AT compared to control (46, 48, 49). All three studies showed differences between groups, with two studies (48, 49) highlighting an increase in triceps surae muscle activity, despite a decrease in overall force output within the AT group (*d* = 1.4*) (49). This might indicate a reduced efficiency of the plantar flexors to generate force, in relation to AT pathology (48) or could be a consequence of pain inhibition and central factors (25, 57), whereby pain has been shown to reduce the force output and efficiency of the plantar flexors in healthy populations (66). However, these two studies had different methodological approaches, for example one study reported on maximal contractions (48) whereas the other study investigated sub-maximal contractions (49), therefore direct comparison between studies should be conducted with caution. A different study (46) found alterations in the force sharing profile of the triceps surae muscles in patients with AT vs. controls, reporting a reduced contribution of the lateral gastrocnemius muscle to sub-maximal isometric plantar flexion [*d* = − 0.54 (−1.15, 0.08)]. Although, ES is moderate and 95%CIs do cross zero, which should promote caution, even though it is by a small amount (0.08). However, this finding supports results from other studies within this review, which detected changes in triceps surae activation during running (35, 60, 67) and hopping (23) activities in AT. It could be suggested that alterations in the electromyographic profile of the triceps surae muscle unit are apparent across a range of movement tasks, but that the exact nature of these changes and the causal mechanism requires further deliberation. Besides, the methodological quality of these studies is questionable, and future high-quality trials are necessitated.

### 4.6. Potential changes in hip strength for AT

One study (43) reported that isometric maximal strength of the hip abductors, extensors and external rotators was reduced in AT, with deficits ranging from 28.3%–34.2%. These muscles are the key stabilisers of the proximal limb segment, and a weakness could result in a redistribution of force absorption throughout the kinetic chain, perhaps leading to injury. Conflictingly, a different study (47) found that both increased and decreased maximal hip external rotation strength were associated with AT when associated with a range of other biomechanical variables within an interactive statistical model. Again, upon closer inspection of absolute values the deficits were not clinically meaningful with only small and non-significant effects [*d* = − 0.28 (−0.83, 0.27)]. Additionally, a prospective study (32) was also unable to identify AT patients when considering hip abduction and adduction strength. Therefore, whether strength changes at the hip for athletic AT patients are relevant is difficult to conclude based upon evidence within this review. Although, it still would seem sensible to consider these factors within the clinical reasoning process, based upon other biomechanical alterations reported within AT. Overall, it could be concluded that rehabilitative strategies focusing on the restoration of plantar flexor strength, potentially hip strength in movements of extension, external rotation, and abduction, and possibly knee flexor strength should be incorporated within clinical practice when treating athletic AT patients. The exact mechanism by which such interventions benefit pain or function remains unclear.

### 4.7. Reflex activity upregulated in AT

Both spinal and supraspinal reflexes were reported to be upregulated within AT patients affected side compared to controls, but this was only found in a single study (23). This may indicate a protective response of the injured tendon, perhaps mediated by central factors (25–27). Whether the normalization of reflex responses should be targeted with interventions for AT patients, and their relation to pain and function, is a novel area of research requiring further investigation.

### 4.8. Limitations

The risk of bias assessment indicated a large variation between study designs and methodological approaches, meaning the results of this review should be interpreted cautiously and drawing any conclusions based upon the current data is extremely difficult. A key problem identified in many studies was that control groups were not matched to the patient group by age, whereby the control group was substantially younger in a number of cases (32, 34, 46–48, 50, 36, 37, 39–43, 45). This may have affected the amount of time spent training within a participant's individual sport, and/or the duration of AT pathology, which might directly impact the findings compared between groups. Besides, age is purported to be a risk factor in general for the development of tendinopathy (68). Data for training duration was only reported in five out of the twenty studies (41–43, 47, 48), making such comparisons difficult to conduct. Fourteen of the twenty studies were determined to have a high risk of bias regarding male vs. female sex inclusion, whereby seven of the studies included substantially more males than females within the study design (32, 36, 37, 42, 46, 47, 50) and a further seven studies only included male participants (34, 39, 41, 43, 45, 48, 69). This limits the generalisability of the results to the female population. The bias assessment also identified discrepancies in the symptomatic behaviour of patients within the AT group across studies. Nine of the included studies investigated patients presenting with current symptoms of pain (34–41, 45), whereas the other eleven studies only included AT patients who were currently without symptoms and in a period of remission. The effects of pain on motor behaviour are well documented (23, 25, 46), and should be considered when interpreting findings of the included studies. Finally, the included studies used a wide range of protocols to investigate parameters of gait, joint strength, and other movement behaviours. For example, some participants ran shod (34–40, 42) whilst others were barefoot (32, 33, 41). Moreover, various studies allowed running at a self-selected speed (32, 35, 36, 38–40, 42, 60) while others standardised a specific speed for all participants (33, 37, 41). Such variation in methodologies makes comparisons and discernment of concrete conclusions challenging. As a final point on the design of the included studies, the statistical reporting was not clear in many experiments (see Table 3) and several biomechanical variables were often tested for statistical significance on a single population. This might raise the chance of finding statistical significance by chance alone, and future studies should be designed with appropriate statistical models and be adequately powered. There are also some limitations to be acknowledged related to the methods of this review. Strict inclusion criteria were applied e.g., athletic population, AT diagnosis, healthy control group, meaning a large body of literature regarding AT and biomechanics could not be included for synthesis, and this research is cited here for transparency (63, 70–84). The main reasons for excluding these studies were the study of a non-athletic population or because relevant information could not be obtained from the authors. Whilst a limitation to some extent, this is also inherent to the strength of the study design. Studies were excluded so that a specific athletic population could be considered, in experiments which investigated AT patients compared to healthy control groups as opposed to the contralateral limb. Given research indicating sensory and motor deficits on the contralateral limb and altered pain processing within AT patients (25, 27) this approach seems justified, and potentially more effective in identifying biomechanical alterations or impairments within the AT population. Additionally, for two studies (43, 48) effect sizes couldn’t be calculated as the data was unsuitable or unavailable.

### 4.9. Conclusions

According to evidence synthesised in this review, there appear to be notable biomechanical alterations during a range of movement tasks in athletic populations with AT compared to their healthy control group counterparts. Equally said, there were several biomechanical variables investigated that were not associated with AT, and in general the study quality of the included trials was poor. This is in agreement with other reviews of research in this area that investigated mixed athletic and general populations (2, 10, 20, 21). Having addressed several of the postulated theories regarding habitual motor patterns and their relationship with AT in this review, the authors would find it very difficult to either accept or refute their relevance based upon the current evidence, especially for those related to running gait kinematics. In summary, the proposed alterations include changes in kinematics and muscle activity of the hip and ankle joint during running, alterations in lower limb function during jumping/hopping, strength deficits of the plantar flexors, the knee flexors and possibly the hip joint, and weak evidence for up-regulated reflex activity. It seems logical to conclude that these alterations might form potential treatment targets for clinical interventions, for example strengthening programs for the kinetic chain of the entire lower limb with particular emphasis on the plantar flexors, knee flexors and hip, gait re-training, plyometrics to restore the stretch shortening capacity of the musculotendon unit, and possibly sensory motor training. However, much more research is required in longitudinal study designs before any concrete conclusions can be drawn from the data within this review. Additionally, the effectiveness and exact mechanisms of improvement with such interventions necessitates further research, and these treatments should be applied on an individual basis with consideration of the specific needs of each patient. It should also be emphasised that the biomechanical profile of Achilles tendinopathy patients is likely to be one of many contributing factors to the overall clinical picture, whereby other factors such as training load management, genetics, previous musculoskeletal injuries, cardiometabolic profile, BMI, psychosocial factors, and other co-morbidities, should also be considered. Although, one might expect factors such as training load, previous musculoskeletal injuries, and biomechanics to play a larger role in athletic populations. Future high quality prospective studies are required to explore the causal mechanisms of AT onset and its relation to biomechanics in athletic groups. Until such studies are conducted, it is very difficult to ascertain whether biomechanical variables are the cause or consequence of musculoskeletal injuries such as AT. The altered biomechanical variables reported in this review, could serve as a good starting point for the focus of such research investigations. If future high-quality trials can confirm these alterations, then clinicians might utilise these as clinical markers in the prevention and rehabilitation of Achilles tendinopathy. However, for the time being, caution is very much warranted and there are no solid conclusions that can be drawn based upon the evidence within this review, due to the reported low-quality of the research and paucity of investigations.

## Data Availability

The original contributions presented in the study are included in the article/**Supplementary Material**, further inquiries can be directed to the corresponding author.
